# Biomarkers for Diagnosis and Prediction of Outcomes in Contrast-Induced Nephropathy

**DOI:** 10.1155/2020/8568139

**Published:** 2020-01-24

**Authors:** Justor Banda, Raquel Duarte, Therese Dix-Peek, Caroline Dickens, Pravin Manga, Saraladevi Naicker

**Affiliations:** ^1^Department of Internal Medicine, Faculty of Health Sciences, University of the Witwatersrand, Johannesburg, South Africa; ^2^Ndola Teaching Hospital, Ministry of Health, Lusaka, Zambia; ^3^Division of Cardiology, Charlotte Maxeke Johannesburg Academic Hospital, Johannesburg, South Africa

## Abstract

**Background:**

Serum creatinine is suboptimal as a biomarker in the early diagnosis of contrast-induced nephropathy (CIN). In this study, we investigated a panel of novel biomarkers in the early diagnosis of CIN and in assessing patient outcomes.

**Methods:**

This single-centre, nested, prospective case-controlled study included 30 patients with CIN and 60 matched controls. Serum and urine samples were collected before contrast administration and at 24 hours, 48 hours, and ≥5 days after contrast administration. Concentrations of NGAL, cystatin C, *β*_2_M, IL18, IL10, KIM1, and TNF*α* were determined using Luminex and ELISA assays. Outcomes were biomarker diagnostic discrimination performance for CIN and mortality after generation of area under receiver operating characteristic curves (AUROCs).

**Results:**

Median serum levels for 24 h cystatin C (*p* < 0.01) and 48 h *β*_2_M levels (*p* < 0.001) and baseline urine NGAL (*p*=0.02) were higher in CIN patients compared to controls with AUROCs of 0.75, 0.78, and 0.74, respectively, for the early diagnosis of CIN. Serum *β*_2_M levels were higher in CIN patients at all time points. Elevated baseline serum concentrations of IL18 (*p* < 0.001), *β*_2_M (*p*=0.04), TNF*α* (*p* < 0.001), and baseline urine KIM (*p*=0.01) and 24 h urine NGAL (*p*=0.02) were significantly associated with mortality. Baseline serum concentrations of IL18, *β*_2_M, and TNF*α* showed the best discrimination performance for mortality with AUROCs, all >0.80. Baseline NGAL was superior for excluding patients at risk for CIN, with positive and negative predictive ranges of 0.50–0.55 and 0.81–0.88, respectively. Cystatin C (*p*=0.003) and *β*_2_M (*p*=0.03) at 24 h independently predicted CIN risk. *β*_2_M predicted increased mortality of 40% at baseline and 50% at 24 hours.

**Conclusion:**

Serum cystatin C at 24 h was the best biomarker for CIN diagnosis, while baseline levels of serum IL18, *β*_2_M, and TNF*α* were best for predicting prognosis.

## 1. Introduction

Despite increased morbidity and mortality linked with iodinated contrast media-induced nephropathy (CIN) [1–4], early interventions are delayed due to the suboptimal sensitivity and specificity of serum creatinine in the early diagnosis of CIN [5–12]. Early diagnostic criteria should identify almost 80% of subclinical kidney injury caused by contrast media within 24 hours [5].

Previous studies have demonstrated that neutrophil gelatinase lipocalin-2 (NGAL), cystatin C, interleukin 18 (IL18), kidney injury molecule 1 (KIM1), and beta-2 microglobulin (*β*_2_M) are more sensitive early biomarkers of acute kidney injury (AKI) compared with serum creatinine [10, 12–17] and are better at predicting adverse clinical outcomes [18–20]. Biomarkers are needed for early identification of subclinical AKI, characterised by small increases in levels of serum creatinine [21], as previous studies have shown that even a small increase in serum creatinine (exceeding 44.3 *μ*mol/l) was linked with a 7-fold increased risk of mortality [10, 22].

Cystatin C and *β*_2_M, with molecular weights of 13 kDa and 11.8 kDa, respectively, are nonglycosylated molecules that are increased in the circulation due to impaired glomerular filtration [17, 23, 24]. Cystatin C, produced by all nucleated cells, functions as an intracellular inhibitor of cysteine peptidase [25, 26], and *β*_2_M is found on major histocompatibility complex class-1 nucleated cells [27]. NGAL, a 25 kDa glycoprotein, is upregulated within 2–4 hours of patients undergoing radio contrast administration [5, 7, 15, 28]. Inflammatory cytokines (including IL18, TNF*α*, and IL10) are upregulated in renal injury or damage [29, 30] and released into plasma and urine [17, 21], making them good biomarker candidates. Limited studies have explored their roles in AKI, particularly CIN.

Previous studies on novel biomarkers focused on homogeneous populations characterised by nonheterogeneous AKI insults (surgery and nonmixed intensive care units), but few were based on radiocontrast studies. Our study investigated the diagnostic potential of NGAL, IL18, cystatin C, *β*_2_M, IL10, KIM1, and TNF*α* in predicting risk for CIN and their role in predicting patient outcomes.

## 2. Materials and Methods

### 2.1. Study Design, Setting, and Population

This nested case-controlled study was performed on a subset of patients undergoing contrast media administration at Charlotte Maxeke Johannesburg Academic Hospital, South Africa, from July 2014 to July 2015, who were followed up for development of CIN in a cohort of 371 patients [31]. CIN was defined according to the European Society of Urogenital Radiology (ESUR) as reported previously [32]. Thirty patients with CIN (with available four time-point sera and urine measurements) and 60 matched controls (who did not develop contrast-induced kidney injury) were recruited consecutively. Inclusion criteria for the study were patients aged above 18 undergoing contrast media administration in the Divisions of Radiology and Cardiology. Patients below 18 years, with pre-existing AKI, end-stage renal disease (ESRD) on renal replacement therapy, prior contrast media administration in the preceding 7 days, and pregnancy were excluded. Controls were matched for race, gender, and age at a case : control ratio of 1 : 2 for all time intervals. The Human Research Ethics Committee (HREC) of the University of the Witwatersrand approved the study, and written informed consent was obtained from all patients.

### 2.2. Study Procedures

Whole blood and urine were collected at four time points: baseline (precontrast) and 24 hours, 48 hours, and 5 to 7 days after contrast administration. Blood and urine samples were centrifuged at 5000 rpm at 4°C for 10 and 2 minutes, respectively (U-32012 Centrifuge, Boeco, Germany), and the sera and urine were stored at −80°C until assayed. Concentrations of IL10, IL18, TNF*α*, NGAL, KIM1, and cystatin C were determined using Magnetic Luminex® Screening Assays (#LXSAHM-3, R&D Systems, Inc., Minneapolis, USA) in accordance with the manufacturer's instructions on the BioPlex™ 200 system (Bio-Rad, Texas, USA). The Bio-Plex manager software, version 5, was used for the determination of concentrations. Serum concentrations of *β*_2_M were determined by an enzyme-linked immunosorbent assay (ELISA) (R&D Systems, Inc.). Serum creatinine was determined using the Jaffe method.

### 2.3. Study Outcomes

The study outcomes were discrimination performance of the novel biomarkers for CIN at different time intervals and in-hospital CIN-associated mortality.

### 2.4. Statistical Analysis

Data analyses were performed with Stata version 13 software (STATA, Inc., Texas). Biomarker characteristics were described as medians and interquartile ranges (IQRs) as values were not normally distributed. Urinary KIM1 levels were below detectable limits in 29% of samples analysed. For these samples, a proxy level of 12.2 pg/ml (the lower level of detection (17.3 pg/ml) divided by the square root of 2) was used. Comparisons of biomarkers with CIN and mortality were determined using the Wilcoxon–Mann–Whitney test. To determine discrimination performance of biomarkers for CIN vs. non-CIN and mortality (overall and CIN + mortality), area under receiver operating characteristic curves (AUROCs) were constructed. Sensitivity and specificity were calculated for each point on the curve, and the optimal cutoff point was determined by finding the point with the maximum Youden index (Youden index = sensitivity + specificity − 1). Positive predictive values (PPVs) were calculated for the optimal cutoff point using the following formula: PPV = (number of true positives)/(number of true positives + number of false positives). Similarly, the negative predictive values (NPVs) were calculated for the optimal cutoff point using the following formula: NPV = (number of true negatives)/ (number of true negatives + number of false negatives). Multivariable regression analysis of biomarkers, adjusted for age and gender, were performed to determine predictors of CIN and mortality. *p* values of <0.05 were required for statistical significance.

## 3. Results

### 3.1. Biomarker Characteristics in CIN Patients

This nested study included 30 CIN participants matched with 60 controls with a median age of 50 years (36–61). Participants' demographic characteristics are presented in Table 1. There were no statistical differences in baseline serum creatinine and estimated glomerular filtration rates between the CIN+ and CIN− groups. Of the 7 CIN mortalities (Table 1), 4 (57%) had underlying malignancy, 2 (28%) liver disease, and 1 (14%) sepsis.  Table 2 shows serum and urine biomarker measurements at various time points. Compared to controls, the CIN patients showed increased levels of serum *β*_2_M at all time points and baseline urine NGAL concentrations. Median serum cystatin C was also significantly increased at 24 and 48 h time points in the CIN group.

### 3.2. Diagnostic Accuracy of Biomarkers in Predicting CIN

**Figure 1:**
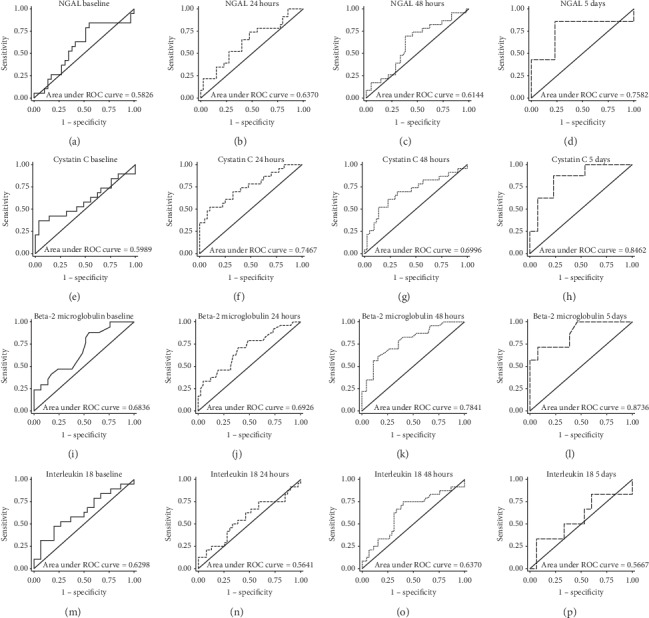
Receiver operating characteristic curves for serum biomarker discrimination performance for CIN at precontrast baseline, 24 hours after contrast administration, 48 hours after contrast administration, and 5 days after contrast administration.

**Figure 2:**
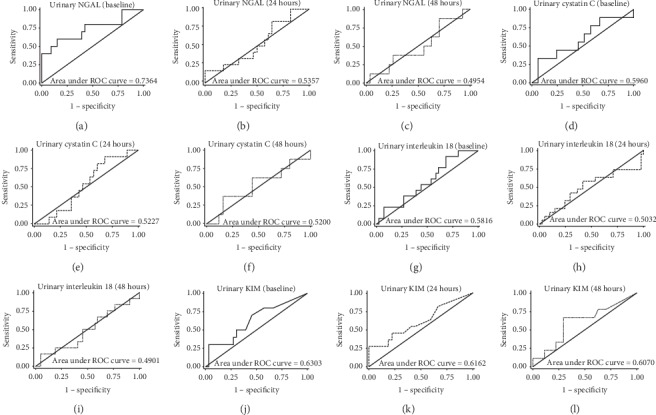
Receiver operating characteristic (ROC) curves demonstrating the ability of urine biomarkers to predict CIN at baseline (precontrast) and 24 hours and 48 hours after radiocontrast administration.

The ROCs for various biomarkers were generated for the determination of early CIN diagnosis (Figures 1 and 2). Serum cystatin C at 24 hours and *β*_2_M at 48 hours and baseline urine NGAL showed the best early discrimination performance for CIN diagnosis with AUROCs of 0.75, 0.78, and 0.74, respectively. Optimal cutoff values for biomarkers in predicting the development of CIN are shown in Table 3.

Baseline serum levels of NGAL and *β*_2_M showed superiority in excluding patients at risk of developing CIN (NPVs > 0.80). Cystatin C at baseline and at 24 hours showed the best predictive values for CIN. Multivariate analysis showed that, after adjusting for age and gender, levels of cystatin C and *β*_2_M at 24 hours showed significant odds ratio (OR = 1.00 (*p*=0.003) and OR = 1.26 (*p*=0.029), respectively) for predicting CIN development.

### 3.3. Diagnostic Accuracy of Biomarkers in Predicting Mortality

**Figure 3:**
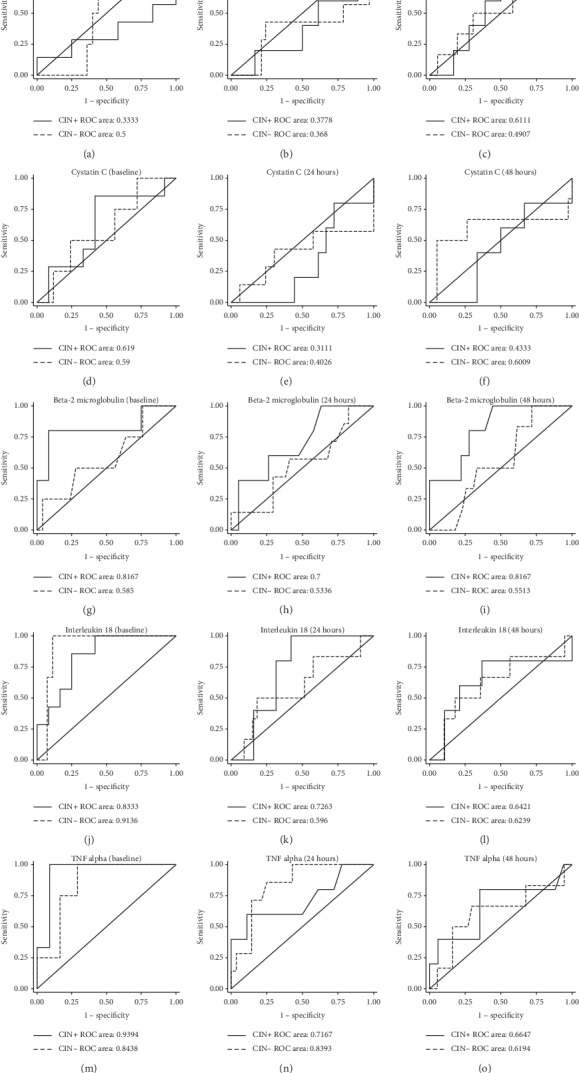
Receiver operating characteristic (ROC) curves demonstrating the ability of serum biomarkers to predict mortality in patients who developed CIN (CIN+; solid line) and those who did not (CIN−; dashed line). Curves are shown for baseline (precontrast) and 24 hours and 48 hours after radiocontrast administration. AUCs for both the CIN+ and CIN− curves are indicated.

**Figure 4:**
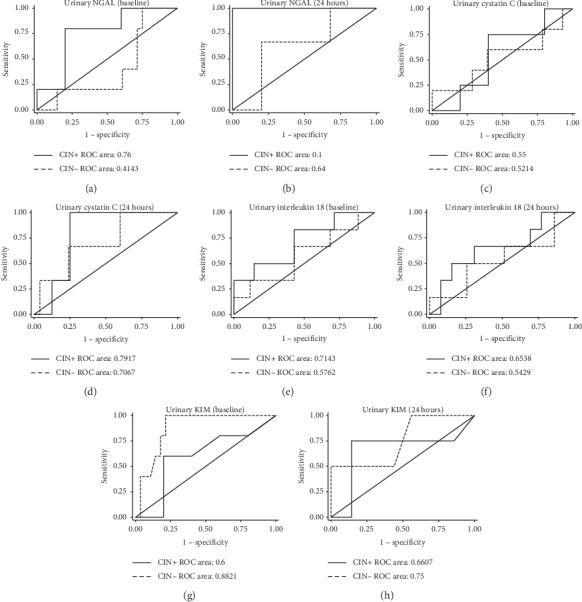
Receiver operating characteristic (ROC) curves demonstrating the ability of urine biomarkers to predict mortality in patients who developed CIN (CIN+; solid line) and those who did not (CIN−; dashed line). Curves are shown for baseline (precontrast) and 24 hours after radiocontrast administration. AUCs for both the CIN+ and CIN− curves are indicated.

Table 4 shows urine and serum biomarker measurements in the patients who survived and in those who died. Baseline serum concentrations of IL18 (*p* < 0.001), *β*_2_M (*p*=0.04), TNF*α* (*p* < 0.001), and baseline urine KIM1 (*p*=0.01) were elevated in the group who died. Twenty-four-hour urine NGAL (*p*=0.02) was also significantly increased in this group. Baseline and postcreatinine measurements were not statistically different between the surviving and nonsurviving groups. ROCs for biomarker discrimination performance for CIN mortality are shown in Figures 3 and 4 for serum and urine biomarkers, respectively. Baseline serum concentrations of TNF*α*, IL18, and *β*_2_M showed best discrimination for CIN mortality with AUROCs of 0.94, 0.83, and 0.82, respectively. Multivariable regression analysis showed baseline and 24-hour *β*_2_M to have significant odds of predicting mortality (OR = 1.41 (*p*=0.01) and OR = 1.51 (*p*=0.003), respectively) after adjusting for other confounders.

## 4. Discussion

This prospective nested case-controlled study confirmed that increased levels of novel biomarkers demonstrated early diagnostic potential for CIN and better negative predictive values for excluding patients at risk of developing CIN. Additionally, increased levels of these biomarkers predicted poor patient outcomes. To our knowledge, it is the first case-controlled study assessing a panel of biomarkers in prediction and prognosis of CIN.

Our study showed that cystatin C at 24 hours after contrast administration showed the best discrimination for CIN and is consistent with previous observational and meta-analysis studies [33–35]. In a recent study of patients undergoing cardiopulmonary bypass surgery with cystatin C measured at 2, 4, 24, and 72 hours after surgery, serum cystatin C levels peaked significantly at 24 hours in the acute kidney injury (AKI) group compared to controls [14]. In this study, cystatin C AUROC at 24-hour discriminating performance for the presence of AKI after surgery in patients with normal baseline renal function was 0.75 [14], a finding very similar to our study.

In another study limited to chronic kidney disease (CKD) patients undergoing angiography, Briguori et al. observed nonsignificant differences between levels of cystatin C in the CIN group compared to controls at baseline [35]. However, 24 hours after contrast media administration, cystatin C was significantly higher in the CIN cases [35]. Their AUROC at 24 hours was ≥0.92, which was higher than in our study [35]. Their study cohort comprised patients with CKD [35], whereas in our study almost 100% of patients had normal baseline renal function. Other studies have shown high AUROCs in CKD patients compared to patients with normal renal function [14].

In a meta-analysis that included 19 studies, Zhang et al. reported that 24-hour cystatin C measurements had the best discrimination for AKI in patients with homogeneous insults to the kidney, and measurements after 24 hours were better in patients with nonhomogeneous insults to the kidney [33]. This meta-analysis revealed that the best AUROCs for AKI were 0.81 at 12 hours after cardiac surgery and 0.92 at 24 hours after contrast media administration [33]. However, this meta-analysis mainly comprised studies of patients with homogeneous insults to the kidney and only included one study on CIN in CKD patients [33].

There are several reasons why serum cystatin C demonstrates best discrimination for detecting early AKI, including CIN. Cystatin C is a filtration biomarker whose serum or plasma concentration correlates linearly with the glomerular filtration rate and therefore is better at detecting subclinical renal function [21]. Cystatin C belongs to the cystatin family and is a small molecule of 13 kDa that inhibits intracellular cystatin peptidases [36] and whose secretion into the circulation is unaffected by muscle mass and volume compared to serum creatinine [34, 36].

In our study cohort, increased levels of *β*2M demonstrated prediction for early diagnosis of CIN and were associated with increased mortality. Several studies on *β*_2_M have focused on populations with underlying CKD [13]. In a study limited to a paediatric population, El-Frargy et al. found significantly increased levels of baseline *β*_2_M in AKI patients compared to controls; however, the levels of serum creatinine remained unchanged [37, 38]. After 72 hours, *β*_2_M demonstrated superiority in detection of AKI with sensitivity and specificity of 98% and 80% vs. 46% and 53% for serum creatinine [37]. In another paediatric study, Herrero-Morín et al. also demonstrated higher levels of *β*_2_M in the AKI group compared to controls despite insignificant changes in levels of serum creatinine [38]. *β*_2_M was also superior in early detection of AKI with an AUROC of 0.80 vs. 0.63 for serum creatinine [38]. This study defined renal disease as glomerular filtration rate (eGFR) <80 ml/min per 1.73 m^2^ [38].

Increased serum *β*_2_M levels were associated with increased mortality in our study. In previous reports among CKD patients who died, increased *β*_2_M was an independent predictor of mortality [39, 40]. Two reasons could explain the association between increased levels of *β*_2_M and mortality in our study cohort. Firstly, the predominant underlying comorbidity of our cohort was malignancy as found in previous studies; malignancy, together with microinflammation, was associated with increased secretion of *β*_2_M [40]. Secondly, in the presence of renal disease including CIN, the impact of high *β*_2_M levels is heightened [40]. Other possible reasons include that *β*_2_M is a filtration biomarker produced by nucleated cells [13], which undergoes almost complete metabolism in the kidney. It is completely absorbed by megalin-mediated endocytosis [13, 27] and is also least affected by extra renal factors [27].

In our study, NGAL was a good predictor for patients at low risk for the development of CIN. This finding is supported by two recent studies limited to CIN: one conducted in Italy among patients with underlying renal disease who underwent angiography [41] and another in Iran conducted in patients with normal renal function [42]. In the Italian study, Quintaville et al. reported NGAL PPV and NPV of 20% and 93%, respectively [41], thus demonstrating the superiority of NGAL in excluding patients at risk for CIN and suboptimal accuracy for diagnosing CIN. In patients with normal renal function undergoing angiography, Khatami et al. reported NGAL positive and negative predictive values of 9.4% and 97.1%, respectively, with suboptimal areas under the receiver operating characteristic curves [42]. Several studies correlating NGAL with the diagnosis of AKI were limited to patients with homogeneous insults to the kidney such as surgical patients [43, 44]. In these studies, early NGAL discrimination for AKI was observed within 2–4 hours after an insult [14, 44]. In a non-CKD population, Schley et al. recently reported a high AUROC of 0.85 in the non-CKD group 4 hours after surgery [14]. However, in patients with CIN, the efficacy of NGAL in discriminating diagnostic performance was conflicting. A recent meta-analysis of 10 studies, limited to NGAL discriminating for CIN, showed variable AUROC [7]. In this meta-analysis, 4 studies looked at patients with CKD [7].

In an additional study, patients undergoing contrast administration were characterised by various heterogeneous insults to the kidney [45] compared to cardiac surgery alone, and additionally, the underlying comorbidities influenced NGAL production. The low diagnostic accuracy for NGAL in CIN could be due to the presence of underlying heterogeneity in kidney function at baseline in these patients and the influence of various other comorbidities [41].

In our study, serum IL18 and TNF*α* together with urine KIM1 demonstrated prognostic significance with mortality in CIN+ patients, despite the poor diagnostic discrimination performance for CIN. In a recent meta-analysis correlating urine IL18 with AKI, the diagnostic discrimination performance of IL18 was modest with an AUROC of 0.66 in adults [46] and was lower compared to other serum biomarkers. Increased IL18 levels are associated with acute tubular necrosis, urinary tract infections, and prerenal failure and therefore may not purely reflect an injury to the kidney [46]. In a previous study, an increase from 25 to 500 pg/ml in urine IL18 at baseline predicted up to 5-fold increased mortality risk [16]. Increased levels of serum IL18 are linked with dysfunction of cardiac myocytes, vascular injury, and apoptosis [47]. In a prospective USA study of hospitalised patients, Liangos et al. reported increased mortality with increasing urine KIM1 levels [48]. KIM1 is a membrane glycoprotein that is expressed by renal tubules and reflects ischaemic and prolonged severe renal injury. Despite its prognostic role, some previous studies have reported urine KIM1 as a suboptimal marker for CIN [49], similar to our study findings. Similar to previous studies [16, 50], our study showed increased levels of serum IL18 in the nonsurviving group.

The strength of this study is that it was a prospective case-controlled study that evaluated serum and urine biomarkers and also compared several biomarkers in discriminating CIN diagnosis and patient outcomes. Additionally, our study population was heterogeneous unlike previous homogeneous studies.

The limitation of our study is that it was conducted at a single-centre tertiary hospital. An additional limitation was the inability to perform measurements within 2–6 hours after contrast administration which remained a challenge in our study cohort, and few patients in our study had intra-arterial contrast media administration unlike previous studies.

In conclusion, novel biomarkers have better diagnostic discrimination for CIN and prediction of outcomes in patients with heterogeneous insults to the kidney. Serum cystatin C at 24 h was the best biomarker for CIN diagnosis, while baseline levels of serum IL18, *β*_2_M, and TNF*α* were best for predicting prognosis. However, more studies are needed to explore the impact of late biomarker measurements on CIN and mortality.

## Figures and Tables

**Table 1:** Participants' demographic and clinical characteristics.

Characteristic		CIN+ (30)	CIN− (60)	*p* value
Age (years), median		56.5 (41–62.5)	47 (34.5–60.5)	0.19
Gender Male, *n* (%)		17 (34.6)	32 (65.3)	0.82
Female, *n* (%)		13 (31.7)	28 (682)	
Hypertension, *n* (%)	Yes	9 (47.3)	10 (52.6)	0.18
	No	21 (29.5)	50 (70.4)	
Diabetes mellitus, *n* (%)	Yes	4 (33.3)	8 (66.7)	0.62
	No	26 (33.3)	52 (66.7)	
Cancer, *n* (%)	Yes	11 (30.5)	25 (69.4)	0.82
	No	19 (35.1)	35 (64.8)	
HIV positive, *n* (%)	Yes	6 (27.3)	16 (72.7)	0.41
	No	24 (35.3)	44 (64.7)	
Baseline urea (mmol/L), median		4.9 (3.9–7.7)	4.1 (3.3–6.2)	0.19
Baseline creatinine (*μ*mol/L), median		69 (53–96)	67 (52–84.5)	0.69
Baseline eGFR (ml/min/1.73 m^2^), median		107 (72–133)	113 (88–136)	0.47
Baseline eGFR <60 ml/min/1.73 m^2^, *n* (%)	Yes	2 (50.0)	2 (50.0)	0.59
	No	28 (32.5)	58 (67.4)	
Postcreatinine (*μ*mol/L), median		104 (85–156)	63.5 (46.5–76.5)	<0.001
Serum albumin (g/dL), mean		33.6 (SD 7.6)	36.6 (SD 7.0)	0.07
Haemoglobin (g/dL), mean		11 (SD 2.9)	12.1 (SD 2.4)	0.06
Diastolic blood pressure (mmHg), median		72 (66–84)	73 (67–83)	0.76
Systolic blood pressure (mmHg), mean		116.3 (SD 15.9)	119.7 (SD 14.4)	0.37
Type of procedure, *n* (%)	IV	26 (33.1)	51 (66.2)	0.53
	Arterial	4 (30.7)	9 (69.2)	
Duration (days), median		16.5 (10–23)	12 (9–21)	0.42
Mortality, *n* (%)	Yes	7 (46.7)	8 (53.3)	0.24
	No	23 (30.7)	52 (69.3)	

HIV, human immunodeficiency virus; eGFR, estimated glomerular filtration rate; CIN, contrast-induced nephropathy; SD, standard deviation.

**Table 2:** Biomarker characteristics in CIN+ and CIN− participants at various time points.

Variable	CIN+ (*n* = 30)	CIN− (*n* = 60)	*p* value
sNGAL_p (ng/ml)	100.31 (64.28–142.01)	74.33 (43.97–127.99)	0.34
sNGAL_24 (ng/ml)	99.61 (72.26–135.98)	78.42 (51.12–107.00)	0.07
sNGAL_48 (ng/ml)	83.81 (57.90–109.05)	60.91 (37.36–100.71)	0.13
sNGAL_5 (ng/ml)	96.20 (74.48–156.66)	65.77 (51.94–72.44)	0.06
uNGAL_p (ng/ml)	88.4 (39.3–366.2)	34.1 (17.2–62.2)	0.02
uNGAL_24 (ng/ml)	40.1 (33.1–96.9)	46.5 (28.5–100.6)	0.72
uNGAL_48 (ng/ml)	39.4 (22.1–100.9)	49.5 (14.2–98.3)	0.96
sCystatin C_ p (ng/ml)	711.45 (550.08–934.10)	687.41 (566.61–769.76)	0.25
sCystatin C_24 (ng/ml)	856.59 (620.75–1002.96)	617.42 (533.11–805.20)	<0.01
sCystatin C_48 (ng/ml)	764.32 (560.28–1010.71)	572.13 (461.67–708.11)	0.01
sCystatin C_5 (ng/ml)	811.52 (708.54–986.12)	596.14 (534.56–684.38)	0.01
uCystatin C_p (ng/ml)	53.7 (32.0–412.1)	49.4 (13.6–170.0)	0.38
uCystatin C_24 (ng/ml)	107.8 (64.3–157.7)	95.9 (27.0–193.9)	0.82
uCystatin C_48 (ng/ml)	47.8 (16.6–166.8)	43.5 (17.7–132.5)	0.87
sIL18_p (pg/ml)	170.41 (105.19–327.4)	123.73 (65.87–178.2)	0.13
sIL18_24 (pg/ml)	152.32 (92.905–279.62)	122.36 (82.45–256.6)	0.40
sIL18_48 (pg/ml)	137.62 (100.965–285.14)	95.75 (73–165.59)	0.06
sIL18_5 (pg/ml)	133.385 (122.36–395.75)	131.93 (70.82–294.91)	0.64
uIL18_p (pg/ml)	102.8 (55.3–185.6)	76.8 (33.2–189.0)	0.38
uIL18_24 (pg/ml)	145.1 (22.6–326.2)	131.0 (50.7–262.4)	0.97
uIL18_48 (pg/ml)	125.1 (50.2–348.7)	124.6 (57.0–372.6)	0.92
s*β*2M_p (*μ*g/ml)	4.4 (3.8–7.8)	3.8 (3.2–4.9)	0.04
s*β*2M_24 (*μ*g/ml)	4.55 (3.9–7.55)	3.7 (2.9–4.8)	0.01
s*β*2M_48 (*μ*g/ml)	5.1 (3.8–6.9)	3.3 (2.7–4.5)	<0.001
s*β*2M_5 (*μ*g/ml)	12.1 (4.4–16.4)	3.7 (3.1–4.9)	0.01
sTNF*α*_p (pg/ml)	4.87 (4.15–9.12)	4.6 (2.65–5.95)	0.12
sTNF*α*_24 (pg/ml)	5.3 (4.15–6.7)	5.23 (3.43–7.39)	0.94
sTNF*α*_48 (pg/ml)	5.9 (4.6–6.7)	4.29 (2.6–7.04)	0.06
sTNF*α*_5 (pg/ml)	4.3 (3.43–5.23)	6.315 (4.26–8.26)	0.22
sIL10_p (pg/ml)	4.94 (4.5–11.3)	4.1 (2.59–5.4)	0.19
sIL10_24 (pg/ml)	4 (3.4–5.5)	3.9 (2.6–5)	0.45
sIL10_48 (pg/ml)	4.6 (3.7–9.2)	3.4 (2–4.5)	0.10
sIL10_5 (pg/ml)	9.5 (9.5–9.5)	4.2 (3.24–19.4)	0.51
uKIM_p (pg/ml)	108.1 (39.9–1593.2)	39.9 (12.2–274.6)	0.21
uKIM_24 (pg/ml)	144.0 (39.9–1343.0)	91.6 (12.2–196.8)	0.26
uKIM_48 (pg/ml)	160.2 (45.8–303.5)	91.6 (12.2–274.5)	0.34

CIN−, CIN absent; CIN+, CIN present; sNGAL and uNGAL, serum and urine neutrophil gelatinase-associated lipocalin; sIL18 and uIL18, serum urine interleukin 18; s*β*2M, serum beta 2 microglobulin; sTNF*α*, serum tumour necrosis factor alpha; uKIM1, urine kidney injury molecule 1 at various time points.

**Table 3:** Optimal cutoff values for biomarkers in predicting CIN.

	Cutoff point	Sensitivity	Specificity	PPV	NPV
sNGAL_p (ng/ml)	63.15	0.84	0.45	0.50	0.81
sNGAL_24 (ng/ml)	80.81	0.74	0.52	0.47	0.78
sNGAL_48 (ng/ml)	72.95	0.70	0.62	0.50	0.79
sCystatin C_p (ng/ml)	893.43	0.37	0.96	0.88	0.70
sCystatin C_24 (ng/ml)	856.59	0.52	0.90	0.75	0.77
sCystatin C_48 (ng/ml)	764.32	0.52	0.86	0.67	0.78
S*β*_2_M_p (*μ*g/ml)	3.6	0.88	0.45	0.48	0.87
S*β*_2_M_24 (*μ*g/ml)	4.3	0.71	0.61	0.51	0.78
S*β*_2_M_48 (*μ*g/ml)	5.1	0.57	0.88	0.72	0.80
IL18_p (pg/ml)	182.0	0.48	0.80	0.60	0.71
IL18_24 (pg/ml)	161.2	0.50	0.67	0.48	0.68
IL18_48 (pg/ml)	116.6	0.75	0.60	0.50	0.81

sNGAL and uNGAL, serum and urine neutrophil gelatinase-associated lipocalin; sIL18 and uIL18, serum and urine interleukin 18; s*β*2M, serum beta 2 microglobulin.

**Table 4:** Biomarker characteristics in surviving and nonsurviving participants.

Variable	Death (*n* = 15)	Survivors (*n* = 75)	*p* value
sNGAL_p (ng/ml)	78.2 (37.0–105.1)	79.4 (57.2–131.5)	0.37
sNGAL_24 (ng/ml)	71.4 (32.8–108.8)	82.7 (58.3–119.3)	0.24
sNGAL_48 (ng/ml)	88.9 (51.3–115.8)	67.3 (41.0–100.7)	0.46
sNGAL_5 (ng/ml)	116.4 (74.4–143.8)	66.1 (43.5–96.2)	0.09
uNGAL_p (ng/ml)	62.9 (19.3–205.8)	36.1 (14.7–68.5)	0.17
uNGAL_24 (ng/ml)	104.7 (85.1–439.1)	36.7 (28.1–72.5)	0.01
uNGAL_48 (ng/ml)	87.6 (38.2–106.7)	44.5 (14.7–96.7)	0.48
sCystatin C_p (ng/ml)	767.38 (633.0–893.4)	670.40 (548.5–789.5)	0.20
sCystatin C_24 (ng/ml)	723.7 (358.9–811.3)	694.3 (569.1–870.3)	0.34
sCystatin C_48 (ng/ml)	764.3 (240.0–843.3)	603.76 (505.55–760.62)	0.45
sCystatin C_5 (ng/ml)	740.4 (706.2–816.6)	668.1 (550.3–724.6)	0.15
uCystatin C_p (ng/ml)	96.7 (19.0–172.5)	44.9 (13.9–186.5)	0.51
uCystatin C_24 (ng/ml)	163.1 (136.5–238.3)	91.4 (28.1–160.3)	0.10
uCystatin C_48 (ng/ml)	92.1 (44.7–901.5)	40.6 (17.1–134.8)	0.19
sIL18_p (pg/ml)	301.5 (211.1–461.3)	109.0 (64.5–165.5)	<0.001
sIL18_24 (pg/ml)	203.3 (118.8–409.9)	125.2 (77.4–224.1)	0.09
sIL18_48 (pg/ml)	181.67 (86.40–331.40)	110.2 (77.0–156.9)	0.19
sIL18_5 (pg/ml)	356.5 (294.9–395.8)	122.3 (65.0–133.9)	<0.01
uIL18_p (pg/ml)	106.4 (57.7–668.4)	73.5 (33.2–182.2)	0.19
uIL18_24 (pg/ml)	220.9 (49.6–421.2)	131.3 (50.4–256.3)	0.33
uIL18_48 (pg/ml)	163.4 (110.6–638.0)	114.0 (38.1–343.4)	0.06
s*β*2M_p (*μ*g/ml)	7.8 (3.7–9.7)	4.0 (3.2–5.4)	0.04
s*β*2M_24 (*μ*g/ml)	4.6 (3.8–7.4)	4.1 (3.3–5.2)	0.21
s*β*2M_48 (*μ*g/ml)	4.6 (3.0–6.7)	3.7 (2.8–5.0)	0.09
s*β*2M_5 (*μ*g/ml)	8.0 (5.5–16.4)	4.1 (3.1–5.0)	0.03
sTNF*α*_p (pg/ml)	8.2 (6.0–79.6)	4.2 (2.6–5.3)	<0.001
sTNF*α*_24 (pg/ml)	7.40 (5.45–14.69)	4.8 (3.2–5.6)	<0.001
sTNF*α*_48 (pg/ml)	6.31 (3.08–11.37)	4.5 (2.7–6.7)	0.11
sTNF*α*_5 (pg/ml)	8.9 (4.8–10.8)	4.6 (2.7–7.0)	0.02
sIL10_p (pg/ml)	4.9 (3.6–17.8)	4.5 (3.0–5.5)	0.47
sIL10_24 (pg/ml)	15.2 (4.2–33.4)	3.9 (2.9–5.0)	0.08
sIL10_48 (pg/ml)	3.9 (3.0–14.8)	3.6 (2.5–5.0)	0.43
sIL10_5 (pg/ml)	11.3 (3.2–19.4)	4.6 (3.9–9.5)	1.00
uKIM1_p (p/ml)	331.9 (131.6–1476.5)	39.9 (12.2–102.9)	0.01
uKIM1_24 (pg/ml)	712.7 (91.6–1343.0)	91.6 (12.2–183.6)	0.07
uKIM1_48 (pg/ml)	1261.9 (579.1–1605.3)	78.6 (12.2–205.1)	0.01
Base creatinine (*μ*mol/l)	72 (44–96)	68 (55–85)	0.93
Post creatinine (*μ*mol/l)	93 (45–126)	69 (54–91)	0.52

CIN−, CIN absent; CIN+, CIN present; sNGAL and uNGAL, serum and urine neutrophil gelatinase-associated lipocalin; sIL18 and uIL18, serum and urine interleukin 18; s*β*2M, serum beta 2 microglobulin; sTNF*α*, serum tumour necrosis factor alpha; sCystatin C and uCystatin C, serum and urine cystatin C; uKIM1, urine kidney injury molecule 1; *p*, precontrast baseline; 24, 24 hours after contrast administration; 48, 48 hours after contrast administration; 5, 5 days after contrast administration.

## Data Availability

The data utilised to support findings of this study are available from the corresponding author upon request.
